# Emerging WS_2_/montmorillonite composite nanosheets as an efficient hydrophilic photocatalyst for aqueous phase reactions

**DOI:** 10.1038/s41598-019-52191-9

**Published:** 2019-11-08

**Authors:** Kang Peng, Hongjie Wang, Xiaoyu Li, Jianwei Wang, Zhixin Cai, Lei Su, Xingyu Fan

**Affiliations:** 1 State Key Laboratory for Mechanical Behavior of Materials, Xi’an Jiaotong University, Xi’an, 710049 China; 2 School of Materials Science and Engineering, Chang’an University, Xi’an, 710064 China

**Keywords:** Photocatalysis, Two-dimensional materials

## Abstract

Tungsten disulfide (WS_2_) as one of transition metal dichalcogenides exhibits excellent catalytic activity. However, its catalytic performances in aqueous phase reactions are limited by its hydrophobicity. Here, the natural hydrophilic two-dimensional clay was used to enhance the dispersibility of WS_2_ in aqueous phase. WS_2_/montmorillonite (WS_2_/MMT) composite nanosheets were prepared via hydrothermal synthesis of WS_2_ on the surface of montmorillonite from WCl_6_ and CH_3_CSNH_2_. The microstructure and morphology show that WS_2_ nanosheets are assembled parallelly on the montmorillonite with the interface interaction. Through the support of montmorillonite, WS_2_/MMT possesses higher photocatalytic ability for aqueous phase reactions than WS_2_, which could be due to the synergistic effect of higher adsorption property, higher hydrophilicity, dispersibility and more catalytic reaction site. The strategy could provide new ideas for obtaining novel hydrophilic photocatalyst with excellent performance.

## Introduction

Since the discovery of graphene, two dimensional (2D) materials have pioneered a new field for nanomaterials. Due to their specific structure and unconventional physicochemical property, 2D nanomaterials have received world-wide of attention for energy storage and conversion^1^, electronics^2^ and catalysis^3,4^. In addition to graphene, many novel 2D nanomaterials have been found and researched heavily in recent years^5^, such as transition metal dichalcogenide (TMD)^6,7^, layered double hydroxides (LDHs)^8^ and graphene analogues^9,10^. Various 2D composite nanosheets were designed and fabricated via multifarious methods^11^, which exhibit exceptional properties and play important roles in many high-tech fields.

Recently, TMD has attracted a lot of attention due to peculiar electrical and optical characteristics and intrinsic semiconducting properties^12^. Interestingly, their electronic band gap transform into direct band gap from indirect band gap, with thickness decreasing to monolayer or few layers from bulk^13^. Tungsten disulfide (WS_2_) with sandwich structure is a kind of TMD composed of multilayered nanosheets^14,15^. WS_2_ sheets possess high absorption for the visible light and excellent photocatalytic activity^16^, which could be used in hydrogen evolution^17^, degradation of dyes^18^ and reduction of nitrophenol^19^. The photocatalytic ability of WS_2_ nanosheets might mainly originate from unsaturated atoms on the surfaces and edges^20,21^. It is reported that the theoretical conduction band and valance band of WS_2_ are −0.06 and 2.27 eV^22^, and WS_2_ has photocatalytic hydrogen production performance^21^. Conventional methods to obtain few-layered WS_2_ sheets include mechanical exfoliation and chemical vapor deposition. Hydrothermal synthesis using precursor of sodium molybdate could obtain fullerene-like WS_2_ nanoparticles, 1D nanotubes and rods, but 2D nanosheets is hard to get in this way. Jieun Yang and co-workers^23^ first report the synthesis of WS_2_/graphene nanosheets by hydrothermal method from tungsten chloride at 265 °C. The WS_2_ nanosheets easily aggregate to reduce catalytic activity, and assembling them on the support materials is one of effective solutions. WS_2_ and most WS_2_ composites are hydrophobicity and poor dispersibility in aqueous phase. Therefore, design of hydrophilic WS_2_ composites is significant for their catalytic ability in aqueous phase.

Montmorillonite (MMT) is one of natural sheet-like clay mineral with excellent hydrophobicity. Its layer structure consists of a central Al-O octahedral sheet and two Si-O tetrahedral sheets, and the unit layer stacks with cation in the interlayer. MMT possesses large special surface area, excellent adsorptive capacity and hydrophobicity, which make it a promising material for pollutants adsorption, catalyst supports^24–27^, wastewater treatment^28,29^, energy storage matrix^30,31^ and drug delivery systems^32^. Based on the special structure and property of MMT, hydrophilic composite nanosheets might be constructed through MMT supporting WS_2_ nanosheets, which could be used as an efficient photocatalyst for aqueous phase reactions. Hazardous wastewaters with organic dye are greatly harmful to the environment^33–37^. Photocatalysis technology is a potential approach to treat these waste waters^38^, and design of the efficient photocatalyst is one of the key procedures^39,40^.

Herein, we first designed and prepared the WS_2_/montmorillonite (WS_2_/MMT) composite nanosheets as a hydrophilic photocatalyst for aqueous phase reactions. WS_2_/MMT was successfully fabricated through facile *in-situ* hydrothermal synthesis of WS_2_ on MMT. The microstructure and morphology were characterized, and the photocatalytic ability for aqueous phase reactions was evaluated by degradation of organic dye. The effect of MMT on catalytic ability was studied, and the possible catalysis mechanism for photocatalytic degradation of RhB were explored and illustrated in detail.

## Results

**Figure 1:**
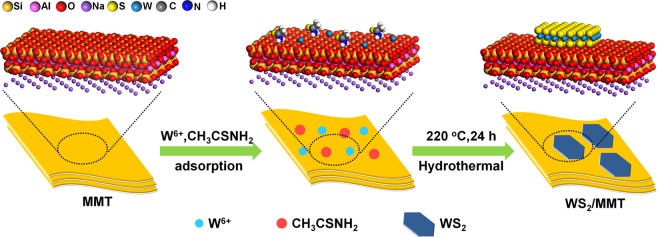
Schematic representation for the hydrothermal preparation of WS_2_/MMT.

As schematically depicted in Fig. 1, the WS_2_/montmorillonite (WS_2_/MMT) composite nanosheets were hydrothermally synthesized with tungsten chloride and thioacetamide at 220 °C for 24 h. Firstly, the montmorillonite (MMT) was added in water solution of WCl_6_ and CH_3_CSNH_2_. The surface of MMT is negatively charged for the Si^4+^ lattice replaced by Al^3+^. Conventional tungstate precursors such as Na_2_WO_4_ and (NH_4_)_10_W_12_O_41_ are unable to adsorb on of MMT. Therefore, WCl_6_ was employed as a precursor of tungsten, and W^6+^ was adsorbed on MMT by electrostatic interaction. In hydrothermal conditions, the thioacetamide was pyrolyzed and released H_2_S, and WCl_6_ was reduced to form WS_2_ by sulfurization. Finally, the WS_2_ nanosheets were nucleated and grown, and the as-prepared WS_2_ was assembled on MMT to prepare WS_2_/MMT nanosheets.

**Figure 2:**
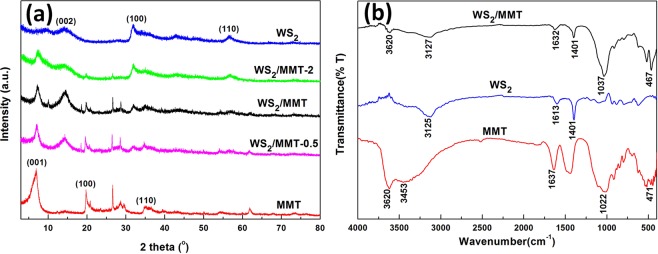
Phase structure and vibrational bands of the samples. (**a**) XRD patterns of MMT, WS_2_, WS_2_/MMT, WS_2_/MMT-0.5 and WS_2_/MMT-2. (**b**) FTIR spectra of MMT, WS_2_ and WS_2_/MMT.

The crystallographic structure was inspected by XRD measurements, and the patterns of MMT, WS_2_, WS_2_/MMT, WS_2_/MMT-0.5 and WS_2_/MMT-2 are presented in Fig. 2a. In the pattern of MMT, the reflection at 7.2° (2*θ*) is attributed to the (001) reflection of Na-montmorillonite, indicating that the *d*_001_ basal spacing is 1.22 nm. The diffraction peaks at 19.7° and 34.7° (2*θ*) are corresponding to (100) and (110) planes of MMT. The diffraction peaks in the pattern of hydrothermally synthesized WS_2_ can be attributed to 2H-WS_2_ phase (JCPDS#08-0237). The reflection at 14.2° due to the (002) diffraction shows the distance of lattice plane along (002) is 0.62 nm for WS_2_ multilayer. The reflections assigned to (100) and (110) planes of WS_2_ are found at 32.7° and 58.4°, respectively. The main reflections of MMT and WS_2_ were observed in XRD pattern of WS_2_/MMT. Compared with WS_2_/MMT, the intensity of the reflections of WS_2_ is relatively lower in the pattern of WS_2_/MMT-0.5, indicating the lower content of WS_2_. The reflection assigned to (100) plane of WS_2_ has higher intensity in the WS_2_/MMT-2, while the diffraction peak corresponding to (002) plane is broader and lower, which could be attributed to lower stacking of WS_2_ layers.

The interface interaction was studied by FTIR analysis. In the FTIR spectrum of MMT (Fig. 2b), the bands due to vibration of Si–O is found at 471 and 1022 cm^−1^, indicating that a silicon-oxygen tetrahedron exists in the layered structure of MMT. The broad band at 3620 cm^−1^ is due to the aluminum hydroxy stretching vibration. The hydroxy bending vibration and H–O–H stretching vibration corresponding to hydrogen bonding water could be observed at 1637 and 3453 cm^−1^. The bands at 1401, 1613 and 3125 cm^−1^ in the FTIR spectrum of WS_2_ are associated with the W–S bending vibration and stretching vibration^41^. For WS_2_/MMT, the bands corresponding to hydroxy decrease significantly, and the band of Si–O shifts to 1037 cm^−1^ from 1022 cm^−1^, which suggests the interface interaction between WS_2_ and MMT.

**Figure 3:**
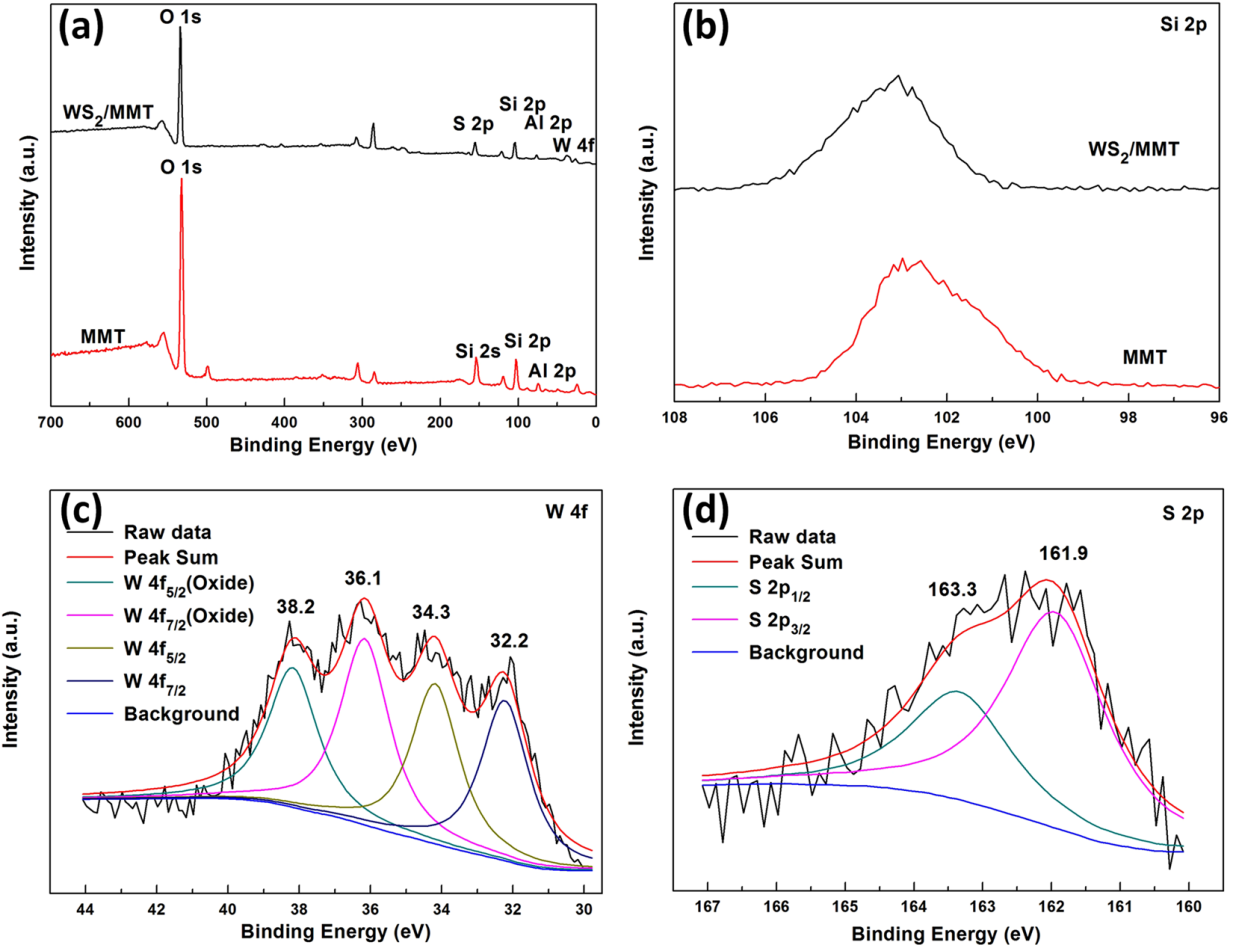
Interface characteristics and chemical status. (**a**) XPS survey spectra and (**b**) high-resolution scans for Si 2p electrons of MMT and WS_2_/MMT, scans for (**c**) W 4f and (**d**) S 2p electrons of WS_2_/MMT.

The chemical statuses of the samples were investigated using XPS. Fig. 3a shows the XPS survey spectra of samples in the range 0–700 eV. Compared with the XPS spectrum survey of MMT, the peaks of W and S are observed in the WS_2_/MMT. The binding energy of Si 2p in WS_2_/MMT (Fig. 3b) shifts to a little higher energy state compared with that of MMT, indicating electronic interaction between WS_2_ and MMT. The W 4 f peaks of WS_2_/MMT (Fig. 3c) are located at the binding energies of 32.2, 34.3, 36.1 and 38.2 eV, respectively. The binding energies of W 4f_7/2_ and W 4f_5/2_ peaks at 32.2 and 34.3 eV are correspond to W^4+^, and the binding energies at 36.1 and 38.2 eV are attributed to W^6+^ (oxide states), which might be due to the partial oxidation of tungsten on the surface and the interface interaction between WS_2_ and MMT. In the scans of S 2p electrons of WS_2_/MMT (Fig. 3d), the binding energies of S 2p_3/2_ and S 2p_1/2_ peaks are 161.9 and 163.3 eV, respectively.

**Figure 4:**
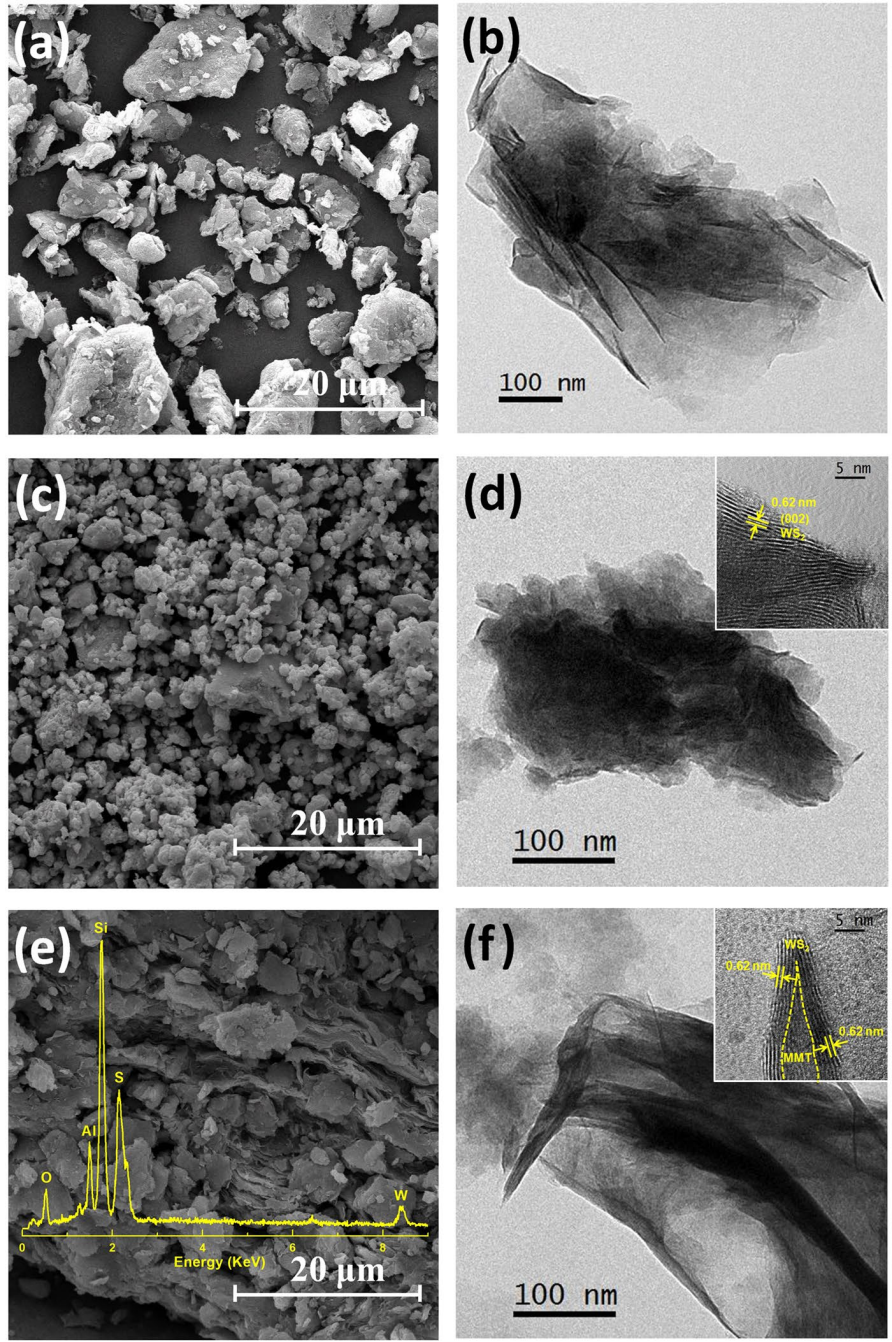
Morphologies of the samples. SEM images of (**a**) MMT, (**c**) WS_2_ and (**e**) WS_2_/MMT and the EDS spectrum. TEM images of (**b**) MMT, (**d**) WS_2_ and (**f**) WS_2_/MMT and the HRTEM image.

The morphologies of samples were characterized with SEM. In the SEM image of MMT (Fig. 4a), the sample presents a lamellar morphology and smooth surface, which is favorable for the supporting of WS_2_. The WS_2_ synthesized by hydrothermal method exhibits the agglomerated particles with irregular shapes (Fig. 4c). For WS_2_/MMT (Fig. 4e), WS_2_ and MMT exhibit two dimensional morphology and stack with each other to form layer structure. In the WS_2_/MMT composites, MMT could reduce the agglomeration of WS_2_. The energy disperse spectrum of WS_2_/MMT indicates that the mass contents of W and S element are 29.84 Wt% and 10.60 Wt%, which is in accord with the theoretical element ratio of WS_2_.

Further details of the microstructure could be obtained by TEM and HRTEM. The TEM image of MMT (Fig. 4b) shows dispersed nanosheet. As shown in Fig. 4d, the synthesized WS_2_ is irregular nanoparticle. The HRTEM image indicates that the (002) plane WS_2_ nanoparticle is 0.62 nm, in accordance with XRD result. From the TEM and HRTEM of WS_2_/MMT (Fig. 4f), the microstructure of sample exhibits composite nanosheets, and WS_2_ nanosheets are assembled parallelly on MMT. The WS_2_ nanosheets could possess better dispersibility and expose more catalytic reaction edges attributed to support of MMT.

**Figure 5:**
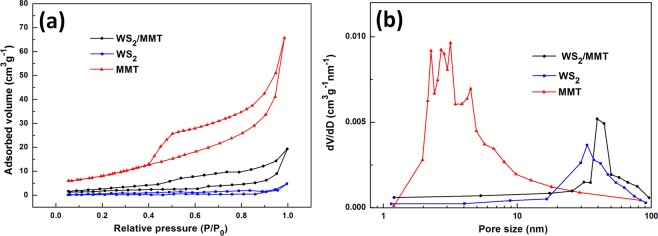
Specific surface area of the samples. (**a**) N_2_ adsorption/desorption isotherm curves and (**b**) pore-size distributions of MMT, WS_2_ and WS_2_/MMT.

The specific surface area was characterized by nitrogen adsorption-desorption isotherms. In the curves of MMT and WS_2_/MMT (Fig. 5a), the type IV adsorption branches are corresponding to the mesoporous structure. The specific surface area of WS_2_/MMT and MMT are calculated to be 16.13 and 38.02 m^2^∙g^−1^, respectively. No hysteresis loop is observed in the curve of WS_2_, and the specific surface area is 6.56 m^2^∙g^−1^. Compared with WS_2_, WS_2_/MMT has relatively higher specific surface area, and it might be because MMT could reduce the stack and inhibit the agglomeration of WS_2_ nanosheets. The pore size distributions are shown in Fig. 5b. The average pore size of MMT is about 3 nm, while those of WS_2_ and WS_2_/MMT are around 40 nm. Due to the support of MMT, WS_2_/MMT composite nanosheets possess larger special surface area than WS_2_, which might provide more reactive sites to enhance photocatalytic activity.

**Figure 6:**
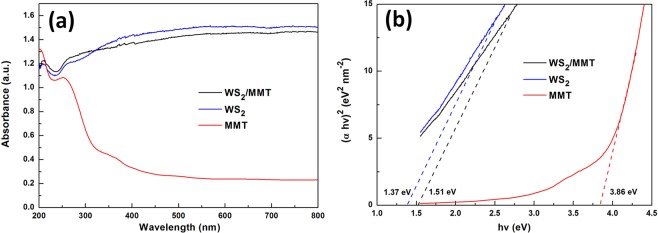
Energy band structure of MMT, WS_2_ and WS_2_/MMT. (**a**) UV-vis diffuse reflectance spectra, (**b**) the corresponding plots of (αhv)^2^ vs. photon energy (hv).

The UV–vis diffuse reflectance spectra of samples are shown in Fig. 6a. The visible light absorption of MMT is very weak. WS_2_ and WS_2_/MMT exhibit considerable visible absorption, which is consistent with the black color of the samples. It is of great significance for visible light photocatalytic application^42^. The band gap energies of MMT, WS_2_ and WS_2_/MMT (Fig. 6b) are 3.86, 1.37 and 1.51 eV, respectively. Compared with WS_2_, WS_2_/MMT possesses larger band gap energy, which could be due to the effects of the few layered WS_2_ and MMT.

Compared with the PL peak of WS_2_ (Fig. S1), the peak shape and position of WS_2_/MMT are similar, while the peak intensity is higher. It might be related to luminescence-inactive multilayer structure in WS_2_. In the PL spectrum of MMT, the peak at 354 and 398 nm could be due to the intrinsic defects of surplus oxygen on the surface and intrinsic diamagnetic defect center, respectively. The PL peak intensity of WS_2_/MMT is weaker than that of MMT, which is attributed to the less recombination of photo-generated carriers.

**Figure 7:**
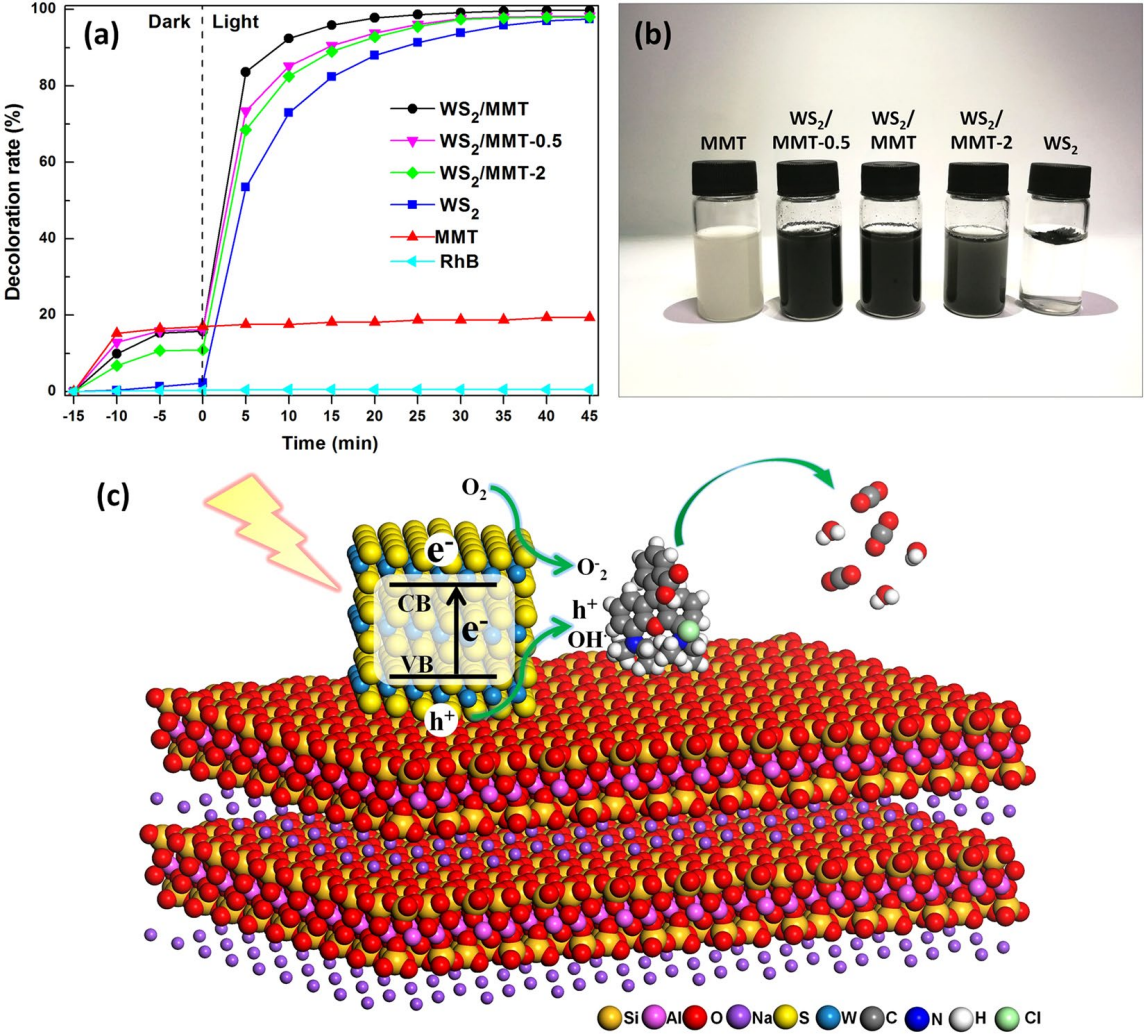
Photocatalytic activity and possible reaction mechanism. (**a**) Decoloration of RhB with MMT, WS_2_, WS_2_/MMT, WS_2_/MMT-0.5 and WS_2_/MMT-2 as catalysts. (**b**) Digital photograph of the samples in aqueous phase. (**c**) Schematic illustration for possible photocatalytic reaction mechanism.

Photodegradation of organic dyes was applied to evaluate the photocatalytic abilities of samples for aqueous phase reactions. The variation of decoloration rate with MMT, WS_2_, WS_2_/MMT, WS_2_/MMT-0.5 and WS_2_/MMT-2 as catalysts is shown in Fig. 7a. The RhB aqueous solution is stable with visible light irradiation during 1 h, and the decoloration rate has hardly the change without catalyst. Adsorption equilibrium was reached without light irradiation for 15 min, and physical adsorption of samples was recorded. MMT has the highest adsorption capacity up to 17.0%, and the adsorption rate of WS_2_ is lowest. The WS_2_/MMT has higher adsorption rate than WS_2_, and the adsorption rate has an increase tendency with the increasing of MMT content. It might be because the special structure of MMT, and the high specific surface area and abundant surface hydroxyl groups are in favor of the adsorption for RhB^43,44^. WS_2_/MMT has the highest degradation speed, and the overall decoloration rate of RhB is up to 99.8% after visible light irradiation for 45 min. The degradation speeds of WS_2_/MMT-0.5 and WS_2_/MMT-2 are higher than that of WS_2_, but lower than WS_2_/MMT. Photocatalytic ability of WS_2_/MMT composite nanosheets is enhanced via the support of MMT. It might be because the MMT sheets prevent WS_2_ nanosheets aggregation, and improve the hydrophilicity and dispersibility of aqueous phase, which could supply composites more reactive sites. As shown in Fig. S4, the photocatalytic ability of WS_2_/MMT does not show obvious change after four cycles, indicating the high stability of WS_2_/MMT in the aqueous phase photocatalytic reaction process. WS_2_/MMT also shows excellent photodegradation performance for Methylene blue (MB), Methyl orange (MO) and Congo red (CR) in the aqueous phase (Fig. S5). The photodegradation of RhB was observed to follow pseudo-first-order kinetics^21^ according to the formula (ln(C/C_0_ = −kt)), where C and C_0_ is the homologous and initial concentration, and k is apparent reaction rate constant. The apparent reaction rate constants of WS_2_/MMT and WS_2_ are 0.16 and 0.09 min^−1^ (Fig. S6), indicating the higher photocatalytic ability of WS_2_/MMT.

In Fig. 7b, WS_2_/MMT composite nanosheets uniformly disperse in water, while WS_2_ almost floated on the aqueous phase, which could be attributed to the hydrophobicity of hydrothermally synthesized WS_2_. Contact angles of MMT, WS_2_/MMT and WS_2_ (Fig. S2) are 18.7°, 32.3° and 54.5°, respectively. With the supporting of MMT, WS_2_/MMT has better hydrophilicity than WS_2_.

## Discussion

As shown in Fig. S3, the UV–vis absorption peak of RhB is located at 554 nm, while no obvious absorption peak is observed after RhB is photodegraded. The possible photocatalysis mechanism for the degradation of RhB is shown in Fig. 7c. With the excitation of visible light, the photoinduced electrons (e^–^) and holes (h^+^) are generated in WS_2_, respectively^45^. hydroxyl groups could capture h^+^ to form hydroxyl radicals (OH^**·**^), which could restrain recombination and improve the photocatalytic ability^46^. The photoexcited e^–^ electrons might induce the O_2_^–^ with O_2_, and these h^+^, OH^**·**^ and O_2_^–^ could photo-oxidize organic molecules RhB.

In conclusion, WS_2_/MMT nanosheets were prepared by the hydrothermal method, which were utilized as an efficient hydrophilic photocatalyst for aqueous phase reactions. Few-layered WS_2_ nanosheets are grown parallelly on MMT, and MMT could reduce the stack and inhibit the agglomeration of WS_2_ nanosheets, which supply composites more catalytic reaction sites. With WS_2_/MMT as photocatalyst, the overall decoloration capacity of RhB was up to 99.8%. Through the support of MMT, WS_2_/MMT possesses high hydrophilicity and dispersibility of aqueous phase, which is conducive to the enhancement of catalytic ability. The WS_2_/MMT composite nanosheets have potential to treat organic waste water. The strategy could provide insights for construction of efficient hydrophilic photocatalyst with excellent activity for environmental treatment.

## Methods

### Materials

The montmorillonite (MMT) used was obtained from Zhejiang Sanding Technology Co. Ltd. (Zhejiang, China). It consisted primarily of MMT (>97%) with minor impurity of quartz. The chemical compositions of MMT were as follows: SiO_2_ 61.5 Wt%, Al_2_O_3_ 19.3 Wt%, MgO 3.5 Wt%, Fe_2_O_3_ 1.4 Wt%, Na_2_O 2.8 Wt%, CaO 2.5 Wt%, K_2_O 0.6 Wt%, and the loss on ignition was approximately 8.4 Wt%. Tungsten chloride (WCl_6_), thioacetamide (CH_3_CSNH_2_) and Rhodamine B were purchased from Sinopharm Chemical Reagent Co. Ltd. All reagents were analytical grade and used without further purification^46^.

### Preparation

The WS_2_/montmorillonite (WS_2_/MMT) composite nanosheets were synthesized by a facile hydrothermal method. In a typical experiment, 1.785 g of tungsten chloride and 3.415 g of thioacetamide were dissolved in 60 mL of deionized water and mechanically stirred for 30 min at room temperature. 1.000 g of MMT was added in the solution, and the mixture suspension was stirred for another 30 min and sonicated for 10 min at room temperature. Then, the suspension was transferred into a 100 mL Teflon-lined stainless steel autoclave, heated up to 220 °C, and kept for 24 h^11^. After cooling naturally, the precipitates were collected by centrifugation, and subsequently washed several times with deionized water. The final products were obtained after drying at 60 °C for 24 h. For comparison, pure WS_2_ samples were synthesized by a similar process, without MMT. Other samples were similarly prepared with different additive amounts of tungsten chloride and thioacetamide. A sample prepared with 0.893 g of tungsten chloride and 1.708 g of thioacetamide was labeled as WS_2_/MMT-0.5. A sample with 3.570 g of tungsten chloride and 6.830 g of thioacetamide was labeled as WS_2_/MMT-2^6^.

### Characterization

Powder X-ray diffraction (XRD) patterns of the samples were obtained on a RIGAKU D/max-2550 PC X-ray diffractometer with Cu Kα radiation (λ = 0.15406 nm) at a scan rate of 0.02°/s^32^. Fourier transform infrared (FTIR) spectra of the samples were obtained between 4000 and 400 cm^−1^ on a Nicolet Nexus 670 FTIR spectrophotometer using KBr pellets. Scanning electron microscopy (SEM) images were obtained with a JEOL JSM-6360LV scanning electron microscope at an accelerating voltage of 5 kV, which equipped with energy dispersive spectrometer (EDS). Transmission electron microscopy (TEM) and high-resolution TEM (HRTEM) were operated with a JEOL JEM-2100F transmission electron microscope at an acceleration voltage of 200 kV^42^. The N_2_ adsorption-desorption isotherms were record at 77 K and analyzed using an ASAP 2020 surface area analyzer. X-ray photoelectron spectroscopy (XPS) measurements were performed using an ESCALAB 250 spectrometer. The UV-vis diffuse reflectance spectra (UV-vis DRS) were obtained with a Shimadzu UV2450 UV-vis spectrophotometer, and barium sulfate was used as reference. The photoluminescence (PL) experiment were conducted on a Hitachi F-4500 fluorescence spectrometer using an excitation wavelength of 254 nm^3^. The contact angles of the samples were measured using the sessile-drop technique using a goniometer (GBX, France).

### Photocatalytic activity evaluation

Photodegradation of Rhodamine B (RhB) was selected as a typical reaction to evaluate the photocatalytic activity of samples for aqueous phase reactions. The light source was a 150 W high pressure mercury lamp with wave length *λ* > 400 nm. In a typical photocatalytic experiment, 100 mg of catalyst was added in 100 mL RhB aqueous solution (0.02 mmol/L). Firstly, the mixture suspension was magnetically stirred in the dark for 15 min to ensure the establishment of an adsorption-desorption equilibrium between the catalyst and RhB aqueous solution. Then, the reaction vessel was positioned with light irradiation, and 1 mL of 3% H_2_O_2_ was added as oxidant to initiate the reaction. About 3 mL of analytical sample was withdrawn from the reaction suspension every 5 min, and the catalyst was removed by a centrifuge. The concentration of the photodegradable compound was monitored by recording the absorbance (*A*) of the clarified solution at 554 nm with an UV-visible spectrophotometer. The decoloration rate (%) was calculated from the formula: decoloration rate (%) = (*A*_0_ − *A*)/*A*_0_ × 100%, where *A*_0_ was the initial absorbance, and *A* was the absorbance at homologous times^25^. The catalyst was centrifuged for the next cycle. Photocatalytic degradation of Methylene blue (MB), Methyl orange (MO) and Congo red (CR) were performed under the similar condition.

## Supplementary information

Supplementary material

## Data Availability

The data that support the findings of this study are available from the corresponding author on reasonable request.
