# Supplementary material for: Emerging WS2/montmorillonite composite nanosheets as an efficient hydrophilic photocatalyst for aqueous phase reactions
Source: Sci Rep. 2019 Nov 8;9:16325. doi: 10.1038/s41598-019-52191-9 (PMC6842000; doi:10.1038/s41598-019-52191-9)
Supplement: Supplementary file 1 — Supplementary material [file 41598_2019_52191_MOESM1_ESM.docx]

**Supplementary material**

**Emerging WS_2_/montmorillonite composite nanosheets as an** **efficient** **hydrophilic photocatalyst** **for aqueous phase reactions**

Kang Peng ^1^, Hongjie Wang ^1*^, Xiaoyu Li ^2^, Jianwei Wang^1^, Zhixin Cai ^1^, Lei Su ^1^, Xingyu Fan ^1^

^1^ *State Key Laboratory for Mechanical Behavior of Materials,* *Xi'an Jiaotong University, Xi'an, 710049, China.*

^2^ *School of Materials Science and Engineering,* *Chang’an University, Xi’an 710064, China*

^*^ *Corresponding author. Email:* *hjwang@xjtu.edu.cn.*


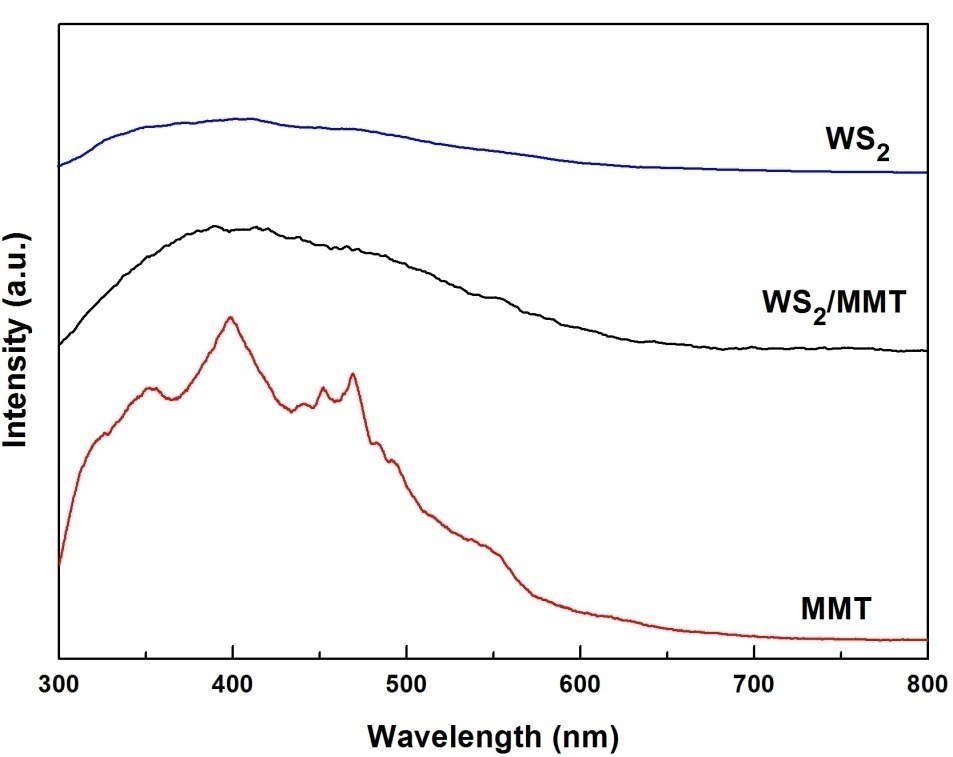


**Figure S1.** The photoluminescence spectra of MMT, WS_2_ and WS_2_/MMT.


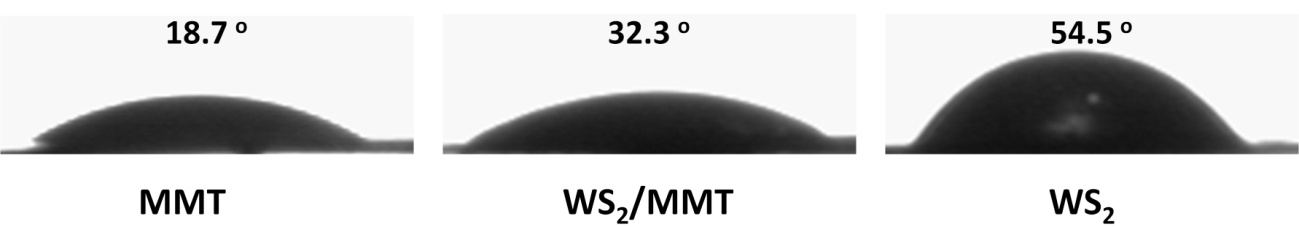


**Figure S2.** Contact angles of water droplets picture collected on the surface of MMT, WS_2_ and WS_2_/MMT.


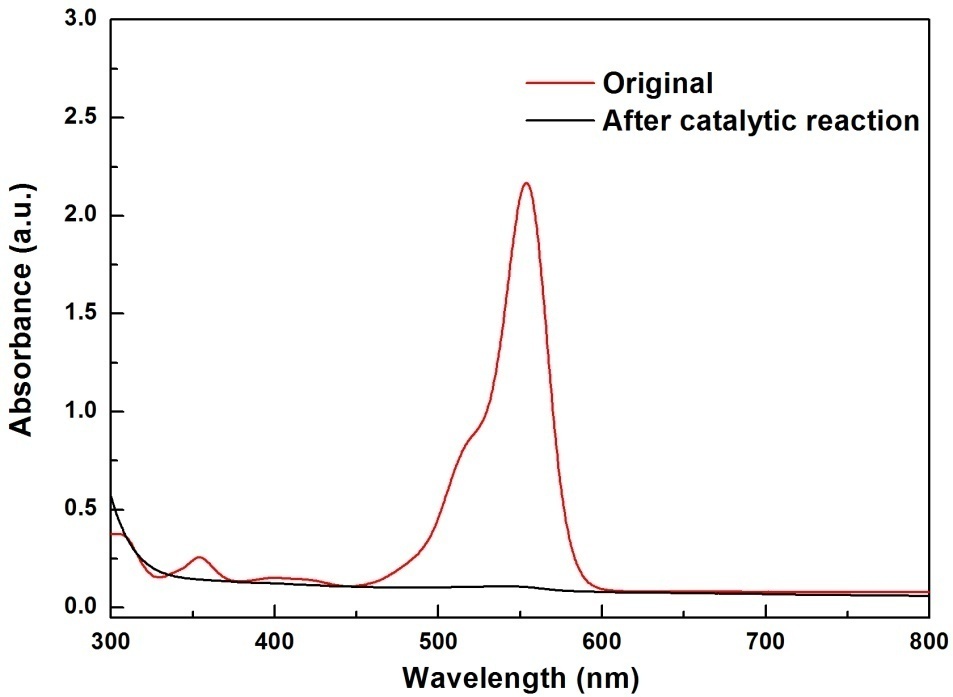


**Figure S3.** The UV–vis absorption spectra of RhB aqueous solution before and after catalytic reaction with WS_2_ /MMT as catalyst.


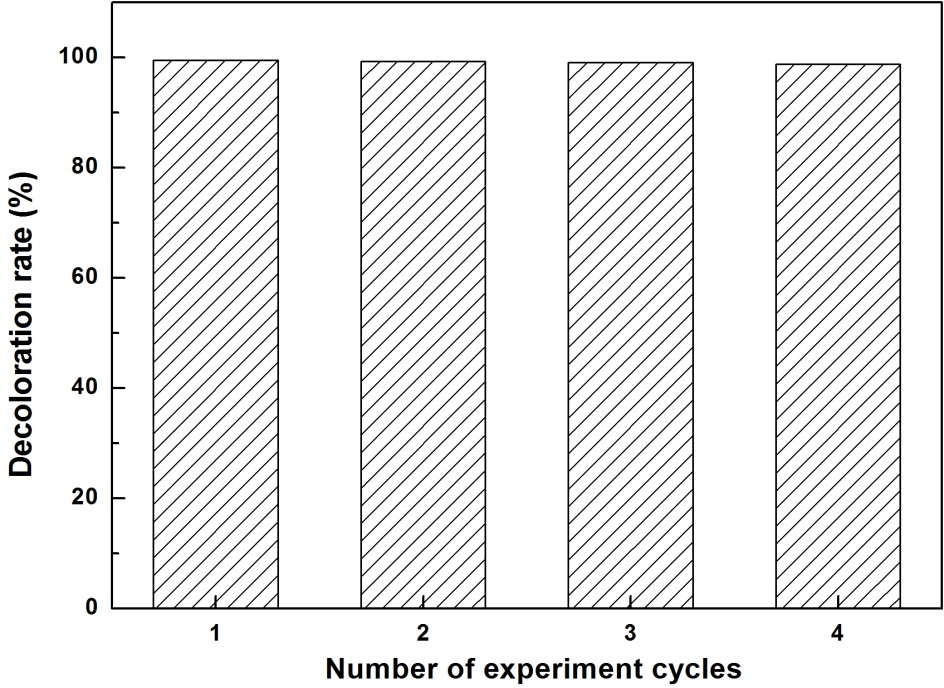


**Figure S4.** Recycling performance of WS_2_/MMT for the decoloration of RhB.


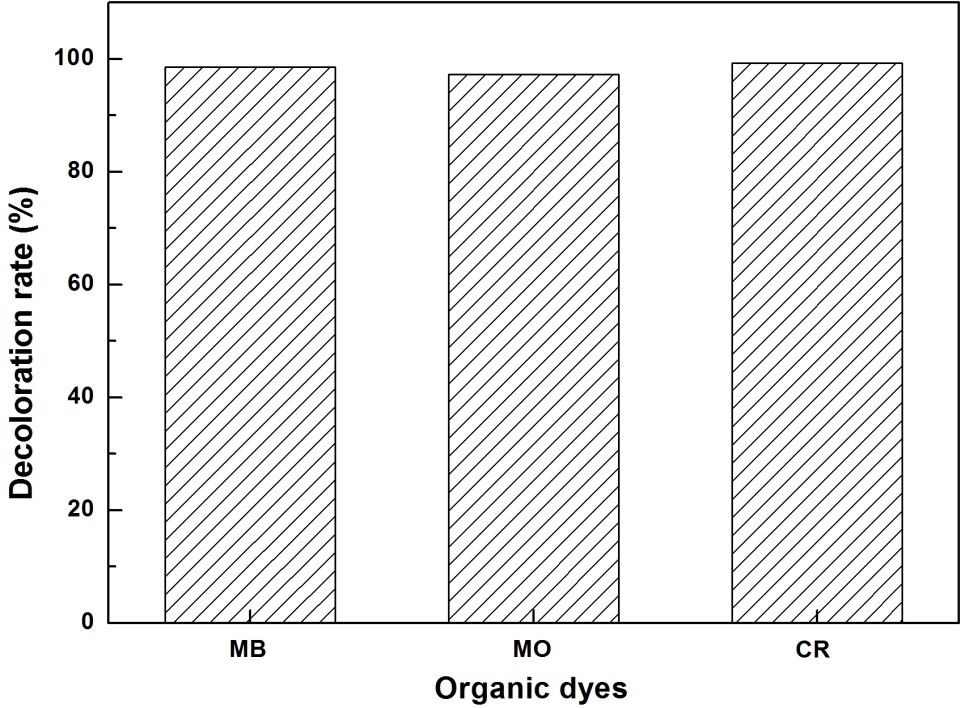


**Figure S5.** Photocatalytic degradation of MB, MO and CR with WS_2_/MMT after light irradiation for 45 min.


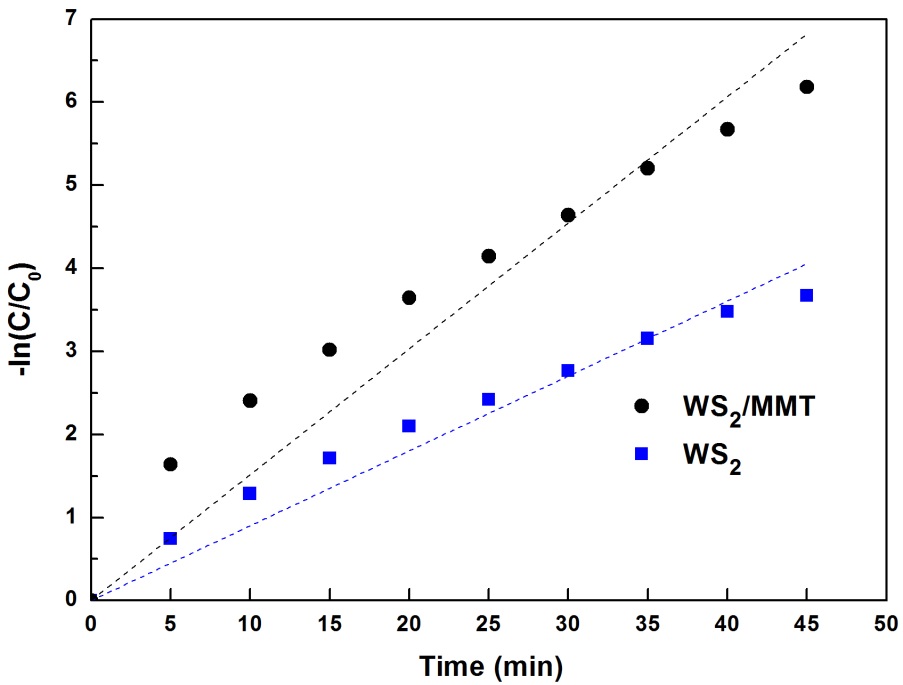


**Figure S6.** Kinetic linear simulation of RhB photocatalytic degradation over WS_2_/MMT and WS_2_.
